# Real-World Adoption of Adjuvant Therapies for Resected Stage IB–III Non-Small-Cell Lung Cancer

**DOI:** 10.3390/cancers17182961

**Published:** 2025-09-10

**Authors:** Arvind Kumar, Steven Tohmasi, Daniel B. Eaton, Nahom Seyoum, Whitney S. Brandt, Theodore S. Thomas, Martin W. Schoen, Nikki E. Rossetti, Su-Hsin Chang, Yan Yan, Mayank R. Patel, Sara Malone, Molly C. Tokaz, Bryan F. Meyers, Benjamin D. Kozower, Varun Puri, Brendan T. Heiden

**Affiliations:** 1 Division of Cardiothoracic Surgery, Department of Surgery, Washington University School of Medicine, St. Louis, MO 63110, USAvarunpuri@wustl.edu (V.P.); 2 Veterans Affairs St. Louis Health Care System, St. Louis, MO 63110, USA; 3 Division of Oncology, Department of Medicine, Washington University School of Medicine, St. Louis, MO 63110, USA; 4 Division of Hematology and Medical Oncology, Department of Internal Medicine, Saint Louis University School of Medicine, St. Louis, MO 63110, USA; 5 Division of Public Health Sciences, Department of Surgery, Washington University School of Medicine, St. Louis, MO 63110, USA; 6 School of Public Health, Washington University in St. Louis, St. Louis, MO 63110, USA; 7 Division of Oncology, Department of Medicine, University of Washington, Seattle, WA 98195, USA

**Keywords:** non-small-cell lung cancer, adjuvant therapy, immunotherapy, targeted therapy, resectable, multimodal treatment

## Abstract

**Background**: Recent clinical trials support adjuvant treatment with novel immunotherapy and targeted therapy agents for selected non-small-cell lung cancer (NSCLC) after resection. The “real-world” implementation of these treatments, however, remains unknown. This study evaluated trends in the uptake of adjuvant chemotherapy, immunotherapy, and targeted therapy for patients with resected NSCLC. **Methods**: Patients with resected stage IB-III NSCLC within the Veterans Health Administration (2017−2023) were included. Use of adjuvant chemotherapy, immunotherapy, and/or targeted therapy was evaluated over the study period. Factors associated with adjuvant therapy use were identified using multivariable-adjusted logistic regression. **Results**: Of the 1980 patients included, 846 (42.7%) underwent adjuvant therapy. There was a modest but not statistically significant increase in adjuvant therapy use from 37.1% in 2017 to 45.9% in 2023. Use of adjuvant chemotherapy alone declined from 36.6% to 23.5%, while use of adjuvant immunotherapy (0.5% to 21.2%) and targeted therapy (0% to 1.2%) increased. Factors associated with adjuvant therapy use included younger age, fewer comorbidities, and higher tumor stage. **Conclusions**: Despite the increased use of adjuvant immunotherapy and targeted therapy for resected stage IB-III NSCLC, overall adjuvant therapy uptake remains low. Further efforts will be necessary to better incorporate these novel treatments into routine clinical practice.

## Simple Summary

Although standard-of-care treatment for high-risk early-stage or locally advanced non-small-cell lung cancer (NSCLC) includes adjuvant chemotherapy following surgical resection, various barriers have been reported that prevent patients from receiving recommended adjuvant treatment. Further addition of adjuvant immunotherapy and molecularly targeted agents has improved short- and long-term outcomes for patients with NSCLC. The results of the present study show that between 2017 and 2023, an increasing proportion of patients with resected stage IB-III NSCLC within the Veterans Health Administration received immunotherapy or targeted adjuvant therapy regimens with or without adjuvant chemotherapy. However, there was an associated decrease in the use of adjuvant chemotherapy alone, such that the overall uptake of adjuvant therapy among these patients did not increase at a comparable rate. These findings highlight that despite the benefits these new treatment regimens may offer, there continue to be underlying challenges with the widespread adoption of adjuvant therapy for NSCLC.

## 1. Introduction

Surgical resection followed by adjuvant systemic therapy has been a standard of care for high-risk early-stage and locally advanced non-small-cell lung cancer (NSCLC) for over 20 years. The International Adjuvant Lung Cancer Trial (IALT), published in 2004, was a multi-institutional trial that compared adjuvant platinum-based chemotherapy versus observation alone for patients with completely resected, non-metastatic NSCLC [1]. This study was the first and largest of its kind to demonstrate significantly higher overall survival among patients who received adjuvant chemotherapy. The IALT also served as the basis for subsequent multi-trial meta-analyses by the Lung Adjuvant Cisplatin Evaluation (LACE) group and NSCLC Meta-analyses Collaborative Group [2,3]. In their analyses, adjuvant chemotherapy was associated with an absolute overall survival benefit of 5.4% (hazard ratio [HR]: 0.89) and 4% (HR: 0.86), respectively. This improvement in overall survival established adjuvant chemotherapy as the recommended standard-of-care treatment for patients who undergo surgical resection for stage II–IIIA NSCLC, especially in the setting of high-risk clinicopathologic features.

More recently, new innovations in multimodal therapy, including immunotherapy and targeted therapy, have revolutionized modern treatment paradigms for NSCLC. On 15 October 2021, the Federal Drug Administration (FDA) approved atezolizumab as adjuvant therapy for patients with completely resected (R0) American Joint Committee on Cancer (AJCC) 7th Edition stage II–IIIA NSCLC after adjuvant platinum-based chemotherapy, based on the results of the phase III randomized clinical trial IMpower010 [4]. This study reported improved disease-free survival for patients receiving atezolizumab compared with adjuvant chemotherapy alone. Similar approval was more recently granted for pembrolizumab as adjuvant therapy for patients with resected stage IB–IIIA NSCLC, who also received adjuvant platinum-based chemotherapy, after survival benefits were demonstrated in the phase III clinical trial KEYNOTE-091/PEARLS [5]. For patients with completely resected, EGFR-mutant, stage IB–IIIA NSCLC, the FDA approved osimertinib as adjuvant therapy with or without chemotherapy on 18 December 2020, based on improved disease-free and overall survival as reported in the phase III clinical trial ADAURA [6].

Despite long-standing data supporting the use of adjuvant therapy after surgery for NSCLC, uptake of adjuvant therapy is inconsistent across patient populations. The ALCHEMIST screening study reported care patterns on 2833 patients with stage IB–IIIA NSCLC from 2014 to 2019 and found that only 57% of patients received any adjuvant chemotherapy [7]. Even in the controlled settings of the recent clinical trials described above, in ADAURA, 60% of patients received adjuvant chemotherapy, which was not required for study inclusion among patients who were randomized [8]. Of the patients in these trials who were randomized to receive adjuvant therapy or placebo, 53% of all patients in ADAURA completed three years of osimertinib or placebo treatment, while 69% of patients in IMpower010 and 58% of patients in Keynote-091/PEARLS completed one year of adjuvant treatment [4,5,6].

Thus far, most data evaluating the guideline-concordant use of adjuvant therapy have only included adjuvant chemotherapy. With the promising long-term benefits of immunotherapy and targeted therapy regimens, understanding the uptake and potential barriers to widespread use of these novel treatments will be essential to ensure patients receive the highest level of care. As such, this study sought to examine the adoption of adjuvant therapy, including immunotherapy and molecular targeted therapy, among patients with resected early-stage and locally advanced NSCLC. Using a uniquely compiled cohort from the Veterans Health Administration database, we evaluated the national trends in uptake of adjuvant therapy, stratified by type of treatment. We then characterized patient- and tumor-related factors associated with the receipt of adjuvant therapy.

## 2. Materials and Methods

### 2.1. Data Source

We conducted a retrospective cohort study of patients treated within the Veterans Health Administration (VHA), leveraging data from the Veterans Affairs Informatics and Computing Infrastructure (VINCI). VINCI provides access to national, patient-level data housed in the Corporate Data Warehouse (CDW), which contains structured information on demographics, diagnoses, procedures, pharmacy dispensing records, and tumor characteristics. To ensure comprehensive data collection with minimal missingness, a dedicated team of research specialists (including one data analyst, two data coordinators, two statisticians, and three physicians) compiled this dataset using a combination of manual chart reviews and natural language processing techniques. The study protocol was approved by the St. Louis VHA Research and Development Committee and deemed exempt from institutional review board approval due to the use of deidentified data (project #1214632, approved 1 October 2019).

### 2.2. Study Design

Patients diagnosed with clinical stage IB–III NSCLC who underwent upfront surgical resection with curative intent between 1 January 2017 and 31 December 2023, were identified using International Classification of Diseases 10th Revision (ICD-10) diagnosis codes, Current Procedural Terminology (CPT) procedure codes, and data from the VHA Oncology Raw and Structured Tumor Tables, as previously described [9]. Staging was defined according to the AJCC 8th edition TNM staging manual [10]. Patients with stage IB-III disease were selected based on current National Comprehensive Cancer Network (NCCN) guidelines that consider surgery followed by adjuvant therapy as an appropriate treatment regimen for this population [11]. This study was limited to patients who underwent complete (R0) resection. Exclusion criteria consisted of undergoing neoadjuvant therapy, pathologic stage IV or unsuspected metastatic disease, and unknown staging or unknown timing of surgery.

The primary outcome of this study was receipt of any adjuvant therapy, stratified by type of treatment. Therapies included platinum-based chemotherapy (cisplatin and/or carboplatin), immune checkpoint inhibitors (durvalumab, atezolizumab, pembrolizumab, or nivolumab), and molecularly targeted therapies (osimertinib). To be considered as having received adjuvant therapy, patients had to receive chemotherapy within 6 months of surgery, or immunotherapy or targeted therapy within 12 months of surgery. Adjuvant therapy modalities were identified using the CDW pharmacy outpatient domain and intravenous table, which provide prescription-level data including drug name, administration route, dose, issue and fill dates, and refills, as well as the Fee basis claim system and consolidated dataset, which derive from claims-level data [12].

Trends in the use of adjuvant therapy were evaluated over the study time period from 2017 to 2023. For this analysis, patients were grouped into three categories stratified by the type of adjuvant therapy they received: (1) adjuvant chemotherapy alone: patients who underwent adjuvant chemotherapy alone, (2) adjuvant immunotherapy: patients who underwent adjuvant immunotherapy with or without chemotherapy, (3) adjuvant targeted therapy: patients who underwent adjuvant targeted therapy with or without chemotherapy.

### 2.3. Statistical Analysis

Sociodemographic data included age at surgery, sex, race/ethnicity (as coded in the CDW and defined per American College of Surgeons Facility Oncology Registry Data Standards), and county ZIP-code-level education and income levels. Comorbidity burden was measured using the Charlson–Deyo Comorbidity Index (CCI), derived from ICD-10 codes recorded in the 36 months preceding surgery [13]. Tumor characteristics included tumor location, tumor histologic subtype, tumor size, and overall stage grouping. Surgical characteristics included extent of resection (wedge, segmentectomy, lobectomy, pneumonectomy), surgical approach (thoracotomy or thoracoscopic [VATS]), and number of lymph nodes evaluated.

Descriptive statistics were used to summarize baseline characteristics and treatment patterns. Categorical variables were compared using Chi-square or Fisher’s exact tests, and continuous variables were compared using Student’s *t*-test or Wilcoxon rank-sum test, as appropriate. Trends in adjuvant therapy use over time were evaluated using the Cochran–Armitage test.

Multivariable-adjusted logistic regression was used to identify factors independently associated with receiving adjuvant therapy, adjusting for the patient, tumor, and treatment covariates described above, which were determined a priori based on clinical relevance and prior literature. Variables were retained in the final model regardless of statistical significance to preserve adjustment for potential confounders.

All analyses were conducted using SAS version 9.3 (SAS Institute, Cary, NC, USA), with statistical significance defined as a two-sided *p*-value < 0.05.

## 3. Results

### 3.1. Study Cohort Characteristics

A total of 1980 patients with resected stage IB–III NSCLC, who met study inclusion criteria, were identified (Table 1). Mean age was 69.9 years (standard deviation [SD]: 6.8). In addition, 96.6% of the cohort was male, and 80.6% was White. Mean CCI was 2.4 (SD: 1.9).

Patients most commonly had tumors located in the right upper lobe (n = 601 [30.4%]) and tumor size between 3.1 and 4 cm (n = 755 [38.1%]). Adenocarcinoma was the most common histologic subtype (n = 850 [45.9%]), followed by squamous cell carcinoma (n = 750 [40.5%]). Most patients had clinical stage IB (n = 640 [32.3%]) or II (n = 1024 [51.7%]) disease, and pathologic stage II (n = 832 [42.0%]) disease. In total, 1393 (70.4%) patients underwent lobectomy, 438 (22.1%) wedge resection, 99 (5.0%) anatomic segmentectomy, and 50 (2.5%) pneumonectomy. VATS was used in 61.5% of cases (n = 1210). The median number of lymph nodes examined was 11 (interquartile range [IQR]: 6, 17).

### 3.2. Use of Adjuvant Therapy

In our study population, 42.7% (n = 846) of patients received any adjuvant therapy. Any adjuvant chemotherapy was used in 41.6% (n = 824) of patients, any adjuvant immunotherapy in 6.5% (n = 129), and any adjuvant targeted therapy in 0.3% (n = 5) (Table 2). Among patients who received any adjuvant immunotherapy, specific immunotherapy agents used included pembrolizumab (n = 56, 43.4%), atezolizumab (n = 33, 25.6%), durvalumab (n = 27, 20.9%), and nivolumab (n = 8, 6.2%).

**Figure 1:**
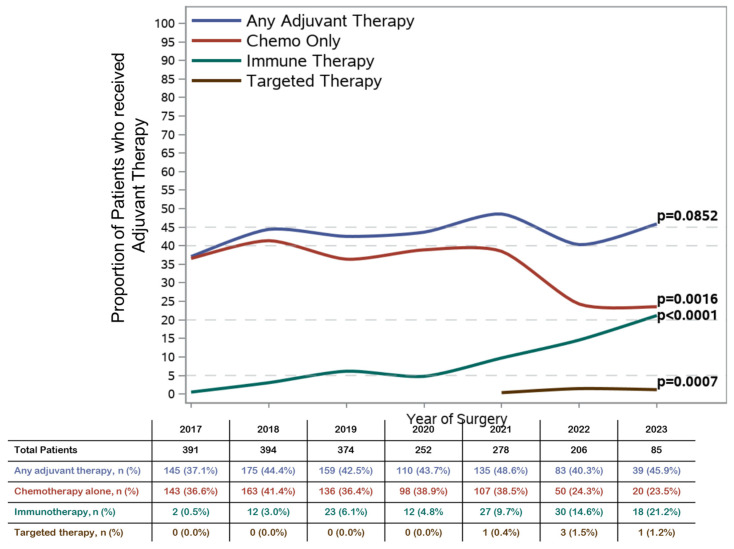
Trends in the receipt of adjuvant therapy, stratified by treatment type, for patients who underwent upfront surgery with completely resected stage IB–III non-small-cell lung cancer between 2017 and 2023.

Over the study period, from 2017 to 2023, there was no significant difference in the overall proportion of patients receiving any adjuvant therapy (37.1% in 2017 vs. 45.9% in 2023, Cochran–Armitage *p* = 0.085) (Figure 1). Among patients who received adjuvant therapy, however, treatment patterns in specific agents substantially evolved. The overall proportion of patients receiving only adjuvant chemotherapy significantly declined from 36.6% in 2017 to 23.5% in 2023 (*p* = 0.0016). In contrast, the use of immunotherapy (with or without chemotherapy) increased from 0.5% in 2017 to 21.2% in 2023 (*p* < 0.0001), and the use of targeted therapy (with or without chemotherapy) showed modest growth, from 0.4% at first appearance in 2021 to 1.2% in 2023 (*p* = 0.0007).

### 3.3. Multivariable Analysis of Factors Associated with Adjuvant Therapy Use

Multivariable-adjusted logistic regression was used to identify factors associated with receipt of any adjuvant treatment following resection of stage IB–III NSCLC (Table 3). Increasing age was associated with decreased odds of receiving adjuvant therapy (odds ratio [OR]: 0.95, 95% confidence interval [CI]: 0.93–0.97). Patients with more comorbidities were less likely to receive adjuvant therapy (OR: 0.91, 95% CI: 0.85–0.96). Sex, race, and county-level education and income levels were not independently associated with adjuvant treatment receipt.

Compared with pathologic stage IB disease, higher pathologic stage was strongly associated with increased odds of receiving adjuvant therapy (Stage II OR: 8.16, 95% CI: 5.58–11.93; Stage III OR: 24.93, 95% CI: 16.10–38.59). Tumor location and tumor histology were not significantly associated with receipt of adjuvant therapy. Patients who underwent VATS rather than thoracotomy had greater odds of receiving adjuvant treatment (OR: 1.34, 95% CI: 1.06–1.70). Extent of lymph node dissection and surgery type were not associated with adjuvant therapy.

## 4. Discussion

In this retrospective cohort study using a highly unique and modern national dataset of patients who underwent upfront surgery for stage IB–III NSCLC between 2017 and 2023, only 42.7% of patients received any adjuvant therapy. There was a modest but not statistically significant increase in the proportion of patients who received any adjuvant therapy from 37.1% in 2017 to 45.9% in 2023. The use of adjuvant immunotherapy (with or without chemotherapy) increased from 0.5% to 21.2%, and the use of adjuvant targeted therapy (with or without chemotherapy) increased from 0% to 1.2% during this time period. In multivariable-adjusted analysis, patient, tumor, and treatment characteristics that were associated with increased use of adjuvant therapy included younger age, fewer comorbidities, higher stage, and surgery via VATS compared with thoracotomy.

This study builds upon the previous literature that has reported a low uptake of adjuvant therapy for patients with resected NSCLC. The ALCHEMIST trial was performed to screen and enroll potentially eligible patients with completely resected stage IB–IIIA NSCLC into multimodal adjuvant therapy trials [7]. Between 2014 and 2019, 2833 patients screened in the study were not subsequently enrolled in a clinical trial, representing a cohort who would have undergone “real-world” treatment protocols. Only 57% of this subgroup received any adjuvant chemotherapy, while 44% received at least four cycles of platinum-based chemotherapy, and 34% received cisplatin-based therapy. Several previous studies have also evaluated the proportion of patients who underwent guideline-concordant adjuvant chemotherapy in the National Cancer Database, reporting adjuvant chemotherapy use in 49−70% of patients with locally advanced NSCLC [14,15,16].

Suboptimal uptake of adjuvant chemotherapy has even been demonstrated within the structured environments of clinical trials, which often include the healthiest and most motivated patients. In the original adjuvant chemotherapy trials included in the LACE meta-analysis, only 59% of patients in the chemotherapy arms received an adequate total dose of cisplatin, while 9% of patients did not receive any chemotherapy [2]. In the randomized phase III clinical trial VIOLET, which compared thoracoscopic and open lobectomy for early-stage NSCLC from 2015 to 2019, and did not include systemic therapy as a component of randomization, 48% of eligible patients received chemotherapy [17]. Similarly, in ADAURA, which did not require patients to undergo adjuvant chemotherapy prior to randomization between osimertinib and best supportive care, 60% of all patients and 76% of patients with stage II–III disease received adjuvant chemotherapy [8].

To our knowledge, this study is the first of its kind to report on national trends in the use of emerging adjuvant therapies, including immunotherapy and targeted therapy, leveraging a very modern dataset from the VHA from 2017 to 2023. These results demonstrated increased use of adjuvant immunotherapy and targeted therapy and an associated decrease in adjuvant chemotherapy alone. This finding likely reflects two of the primary benefits of these novel therapies compared with chemotherapy—more rapid and durable tumor response and fewer treatment-related toxicities [4,5,6,18]. The balance between survival benefit and potential toxicities is a key theme for providers and patients when considering treatment options. In a survey of lung cancer clinicians, Blinman et al. reported that a median of 9 additional months beyond a presumed 5 years of survival, or a 5–10% increase in survival rate, was the minimum survival benefit that participants would consider sufficient to pursue adjuvant therapy for their patients [19]. In a qualitative study of 22 lung cancer patients exploring themes that impacted their decision to undergo adjuvant therapy, patients described potential survival benefit as one of the strongest motivators for treatment [20]. Yet, adverse effects of chemotherapy were considered the most significant hurdle to overcome. As immunotherapy and targeted therapy options continue to offer patients safer and improved short- and long-term outcomes, their increased use demonstrated in our analysis likely represents only the beginning of a widescale shift towards these multimodal treatment protocols.

Despite this increase in novel adjuvant treatments, a second important finding of our study was the limited overall increase in adjuvant therapy use between 2017 and 2023, with a large proportion of patients failing to receive guideline-concordant adjuvant therapy. During the enrollment processes in IMPower010 and PEARLS/Keynote-091, of the patients registered and screened for eligibility, 60−62% were eligible to receive adjuvant immunotherapy after meeting study inclusion criteria, based on receiving surgery with negative margins, having an appropriate performance status, and receiving adjuvant chemotherapy, when required [4,5]. In ADAURA, 28% of patients screened had EGFR-mutated tumors and were eligible for randomization [6]. In a more recently published phase III randomized trial, ALINA, which evaluated the use of adjuvant alectinib in patients with resected stage IB–IIIA NSCLC with ALK-positive mutations, 16% of patients met study inclusion criteria and were eligible for treatment with adjuvant alectinib (or placebo) [21]. In 2023, only 21% and 1.2% of patients in the present study received adjuvant immunotherapy or targeted therapy, respectively. Although the patients represented in our study cohort and those screened for eligibility in the above clinical trials may represent different populations, this difference underscores the large gap that remains between the expected number of patients who should receive adjuvant therapy and the number of patients who have.

The limited overall increase in use of adjuvant therapy also highlights the existence of underlying barriers for patients receiving adjuvant therapy, regardless of the type of therapy. In the present study, higher tumor stage, VATS surgical approach, younger age, and fewer comorbidities were significant predictors of receiving adjuvant therapy. Tumor stage has been consistently shown to be one of the strongest predictors of receiving adjuvant chemotherapy in several previous studies [7,14,15,22,23], while age and comorbidities are often cited as limiting factors due to the impact of potential toxicities or lack of eligibility for treatment [7,14,15,22,23,24,25,26].

The decision to recommend adjuvant therapy to patients also depends upon a strong multidisciplinary partnership between thoracic surgeons, medical oncologists, and radiation oncologists, as well as their collective adoption of novel treatments. There is a commonly reported time-lag of up to 17 years between the publication of research evidence and its subsequent implementation in clinical practice [27]. Prolonged time to adoption of advances in the surgical treatment of NSCLC has been previously witnessed with VATS [28], and now robotic surgery [29]. Furthermore, despite the reported potential rates of early detection and improved long-term outcomes with lung cancer screening, rates of screening within the general population remain low [30,31]. Even the use of immunotherapy and targeted therapy relies upon increased adoption of molecular testing, which has its own challenges in clinical practice [32]. Incorporation of new practices, such as those described above, into treatment guidelines can be one method to improve widespread awareness and adherence. Nevertheless, as our group has previously shown, treatment quality and guideline concordance vary greatly in the surgical treatment of NSCLC [33]. The results of the present study only add to this growing body of literature and help advocate for improvements and consistency in overall quality of care.

This study has several strengths. Notably, it uses a uniquely curated and modern dataset to look at near real-time implementation of novel adjuvant therapies in resected NSCLC. Such data are typically lacking or immature in other national datasets. However, this study also has several limitations. First, due to its retrospective nature, this study may be subject to selection bias and confounding, despite our use of multivariable-adjusted analysis. Second, our study may underrepresent the number of patients who received guideline-concordant therapy. Although adjuvant therapy is considered a standard-of-care therapy option for patients with stage IB–III NSCLC, not all patients with stage IB disease require adjuvant treatment to receive guideline-concordant care. Furthermore, the VHA database does not include data on underlying driver mutations such as PD-L1 and EGFR status to determine which patients would receive immunotherapy or targeted therapy. Thus, it is possible that some patients may have appropriately not received such adjuvant therapies. This inability to account for molecular tumor data serves as a potential source of selection bias, as a portion of our patients may not have been eligible for adjuvant therapy. Nevertheless, as reported in the previous literature, the percentage of patients with EGFR and PD-L1 positivity who would qualify for adjuvant treatment is likely much higher than the reported proportion of patients who received treatment in this study [4,5,6,21].

Third, radiation therapy is a key component in the multimodality treatment of NSCLC. The primary outcome in the present study was the use of adjuvant systemic or targeted therapy, and we did not evaluate the use of adjuvant radiation. Specifically, due to limitations in the VHA dataset, we were unable to evaluate granular data about the specific radiation protocols patients may have received. Post-operative radiation therapy (PORT) was a potential treatment option for patients in our study cohort, especially for those with N2-positive disease. Since the publication of the phase III randomized controlled trial, Lung ART, which found no difference in survival between patients with stage IIIAN2 NSCLC who did and did not undergo adjuvant radiation, however, the number of patients who received adjuvant radiation would be expected to decrease [34]. Further research should investigate how the results of this trial as well as the increased use of adjuvant immunotherapy and targeted therapy regimens impact the use of adjuvant radiation.

Fourth, the VHA offers several unique strengths, including being a nationally representative dataset with granular treatment-related data with high accuracy and minimal missing data. Nevertheless, because of the distinct patient population served by the VHA, this dataset may overrepresent certain subgroups, including men, White patients, and those with higher comorbidity burden and higher rates of smoking, compared with the overall population of patients with NSCLC [35]. That said, our group has previously demonstrated that patterns of care and outcomes are similar between patients being treated in both VHA and non-VHA settings [36]. As more data become available on the use of adjuvant immunotherapy and targeted therapy, further research should be conducted to assess relevant trends in other patient cohorts. Fifth, data on patients who may have received post-operative care outside of the VHA system was not represented in this dataset. As we have previously reported, however, most veterans receive the entirety of their care within the VHA and would therefore be accurately represented in our analysis. Finally, qualitative data on why patients did or did not receive adjuvant therapy was unavailable.

## 5. Conclusions

In conclusion, the results of this national analysis of patients with completely resected stage IB–III NSCLC within the VHA between 2017 and 2023 demonstrate that only 42.7% of patients received any adjuvant therapy. Over the same time period, there was a significant increase in the adoption of molecularly targeted therapies and immunotherapies into clinical practice, with an associated decrease in the use of adjuvant chemotherapy alone. There was no significant change in the total proportion of patients receiving any adjuvant therapy.

Although more patients are pursuing multimodal treatment options with immuno- and targeted therapy, the results of our study highlight that the increased use of these treatment options has not translated to an increase in the overall uptake of adjuvant therapy. Older patients and patients with higher-stage disease or more comorbidities continue to be less likely to receive any type of adjuvant therapy compared with their counterparts. Data from recently published and ongoing clinical trials continue to present improved short- and long-term outcomes for patients with non-metastatic lung cancer after surgery followed by novel adjuvant therapy agents. Further efforts will be necessary to ensure that these emerging therapies are implemented appropriately into routine clinical practice. Equitable and widespread implementation of these therapies to appropriately selected patients who meet guideline- or clinical-pathway-based criteria in the adjuvant setting may disproportionately improve patient outcomes in resectable NSCLC.

## Figures and Tables

**Table 1:** Baseline characteristics of the study population (patients who underwent upfront surgery with completely resected stage IB–III non-small-cell lung cancer).

	Total Cohort (N = 1980)
Age (years), mean (SD)	69.9 (6.8)
Female, n (%)	68 (3.4%)
Race, n (%)	
White	1595 (80.6%)
Black	323 (16.3%)
Other	27 (1.4%)
Unknown	35 (1.8%)
Charlson–Deyo comorbidity index, mean (SD)	2.4 (1.9)
ZIP-code-level high school failure rate, n (%)	
<7%	344 (17.7%)
7−12.9%	624 (32.1%)
13−20.9%	607 (31.2%)
≥21%	372 (19.1%)
ZIP-code-level median income, n (%)	
<USD 38,000	456 (23.4%)
USD 38,000−USD 47,999	511 (26.3%)
USD 48,000−USD 62,999	531 (27.3%)
≥USD 63,000	449 (23.1%)
Tumor location, n (%)	
Right upper lobe	601 (30.4%)
Right middle lobe	78 (4.0%)
Right lower lobe	429 (21.7%)
Left upper lobe	491 (24.8%)
Left lower lobe	321 (16.2%)
Paired	1 (0.1%)
Unknown	59 (3.0%)
Tumor histology, n (%)	
Adenocarcinoma	850 (45.9%)
Squamous	750 (40.5%)
Other	253 (13.7%)
Tumor size (cm), n (%)	
≤1.0	4 (2.2%)
1.1−2.0	182 (9.2%)
2.1−3.0	146 (7.4%)
3.1−4.0	755 (38.1%)
4.1−5.0	385 (19.4%)
5.1−6.0	197 (10.0%)
6.1−7.0	127 (6.4%)
>7.0	117 (5.9%)
Unknown	27 (1.4%)
Surgery type, n (%)	
Lobectomy	1393 (70.4%)
Pneumonectomy	50 (2.5%)
Segmentectomy	99 (5.0%)
Wedge resection	438 (22.1%)
Surgical approach, n (%)	
Open	759 (38.6%)
Thoracoscopic (VATS)	1210 (61.5%)
Lymph nodes evaluated, median (IQR)	11 (6, 17)
Clinical stage, n (%)	
I	640 (32.3%)
II	1024 (51.7%)
III	316 (16.0%)
Pathologic stage, n (%)	
0	6 (0.3%)
I	658 (33.2%)
II	832 (42.0%)
III	391 (19.8%)
Unknown	93 (4.7%)

IQR—interquartile range; SD—standard deviation; VATS—video-assisted thoracoscopic surgery.

**Table 2:** Proportion of patients who received adjuvant therapy after upfront surgery with completely resected stage IB–III non-small-cell lung cancer stratified by treatment type.

	Total Cohort (N = 1980)
Adjuvant Chemotherapy, n (%)	824 (41.6%)
Adjuvant Immunotherapy, n (%)	129 (6.5%)
Atezolizumab, n (%)	33 (25.6%) ^1^
Durvalumab, n (%)	27 (20.9%) ^1^
Nivolumab, n (%)	8 (6.2%) ^1^
Pembrolizumab, n (%)	56 (43.4%) ^1^
Adjuvant Targeted Therapy, n (%)	5 (0.3%)
Osimertinib, n (%)	5 (100.0%) ^2^

^1^ Reported as proportion out of total number of patients who received any adjuvant immunotherapy. ^2^ Reported as proportion out of total number of patients who received any adjuvant targeted therapy.

**Table 3:** Multivariable-adjusted logistic regression evaluating independent predictors of the receipt of adjuvant therapy for patients who underwent upfront surgery with completely resected stage IB–III non-small-cell lung cancer.

	Odds Ratio (OR)	95% CI	*p*
Age (per year)	0.95	0.93, 0.97	<0.001
Female vs. male	0.97	0.53, 1.76	0.91
Race (ref = White)			0.19
Black	1.39	1.00, 1.92	
Other	1.27	0.46, 3.50	
Unknown	1.58	0.65, 3.83	
Charlson–Deyo comorbidity index (per point)	0.91	0.85, 0.96	0.002
ZIP-code-level high school failure rate (ref = <7%)			0.24
7−12.9%	0.76	0.54, 1.09	
13−20.9%	0.69	0.46, 1.03	
≥21%	0.63	0.39, 1.01	
ZIP-code-level median income (ref = <USD 38,000)			0.60
USD 38,000−USD 47,999	0.83	0.59, 1.17	
USD 48,000−USD 62,999	0.92	0.64, 1.33	
USD 63,000+	0.79	0.52, 1.22	
Tumor location (ref = right upper lobe)			0.51
Right middle lobe	0.81	0.45, 1.45	
Right lower lobe	0.85	0.62, 1.17	
Left upper lobe	1.18	0.86, 1.60	
Left lower lobe	1.13	0.80, 1.59	
Paired	N/A	N/A	
Unknown	0.81	0.42, 1.59	
Tumor histology (ref = Adenocarcinoma)			0.11
Squamous	0.93	0.72, 1.19	
Other	1.34	0.95, 1.90	
Tumor size (cm) (ref = ≤1.0)			0.064
1.1−2.0	2.42	1.00, 5.86	
2.1−3.0	1.52	0.62, 3.73	
3.1−4.0	1.88	0.82, 4.33	
4.1−5.0	1.32	0.58, 3.02	
5.1−6.0	1.80	0.76, 4.24	
6.1−7.0	1.75	0.71, 4.23	
>7.0	1.00	0.41, 2.45	
Unknown	1.35	0.38, 4.77	
Surgery type (ref = lobectomy)			0.051
Pneumonectomy	0.91	0.46, 1.82	
Segmentectomy	0.51	0.29, 0.92	
Wedge resection	0.75	0.56, 1.00	
Thoracoscopic (VATS) vs. open	1.34	1.06, 1.70	0.016
Lymph nodes evaluated (per node)	1.00	0.99, 1.01	0.69
Pathologic stage (ref = I)			<0.001
0	3.35	0.54, 20.97	
II	8.16	5.58, 11.93	
III	24.93	16.10, 38.59	
Unknown	15.45	8.29, 28.93	
Year of surgery (per year)	1.08	1.01, 1.15	0.019

CI—confidence interval.

## Data Availability

The data used in this study are maintained by the United States Department of Veterans Affairs (VA), Access Date: 1 January 2025. VA data is available to VA-affiliated researchers with VA-secured computing access after appropriate study protocol approval. For more information, visit https://www.virec.research.va.gov or contact the VA Information Resource Center at VIReC@va.gov. Additional inquiries can be directed to the corresponding author. Deidentified data from the VA study populations discussed in this article can be made available upon request with appropriate Institutional Review Board and VA approval as well as data use agreements. We may balance the potential benefits and risks of each request and then provide the data that can be shared.

## Abbreviations

The following abbreviations are used in this manuscript:

NSCLC	Non-small-cell lung cancer
IALT	International Adjuvant Lung Cancer Trial
LACE	Lung Adjuvant Cisplatin Evaluation
HR	Hazard ratio
FDA	Federal Drug Administration
AJCC	American Joint Committee on Cancer
VHA	Veterans Health Administration
VINCI	Veterans Affairs Informatics and Computing Infrastructure
CDW	Corporate Data Warehouse
ICD	International Classification of Diseases
CPT	Current Procedural Terminology
NCCN	National Comprehensive Cancer Network
CCI	Charlson–Deyo Comorbidity Index
VATS	Video-assisted thoracoscopic surgery
SD	Standard deviation
IQR	Interquartile range
OR	Odds ratio
CI	Confidence interval
PORT	Post-operative radiation therapy
